# The ATX–LPA Axis Regulates Vascular Permeability during Cerebral Ischemic-Reperfusion

**DOI:** 10.3390/ijms23084138

**Published:** 2022-04-08

**Authors:** Susmita Bhattarai, Sudha Sharma, Utsab Subedi, Hosne Ara, Alika Shum, Murov Milena, Md. Shenuarin Bhuiyan, Srivatsan Kidambi, Hong Sun, Sumitra Miriyala, Manikandan Panchatcharam

**Affiliations:** 1 Department of Cellular Biology and Anatomy, Louisiana State University Health Sciences, Shreveport, LA 71103, USA; susmita.bhattarai@lsuhs.edu (S.B.); sudha.sharma@lsuhs.edu (S.S.); utsab.subedi@lsuhs.edu (U.S.); hosne.ara@lsuhs.edu (H.A.); alika.shum@lsuhs.edu (A.S.); milena.murov@lsuhs.edu (M.M.); hong.sun@lsuhs.edu (H.S.); 2 Department of Pathology and Translational Pathobiology, Louisiana State University Health Sciences, Shreveport, LA 71103, USA; shenu.bhuiyan@lsuhs.edu; 3 Department of Chemical and Biomolecular Engineering, University of Nebraska, Lincoln, NB 68588, USA; skidambi2@unl.edu

**Keywords:** lysophosphatidic acid, autotaxin, permeability, ischemic-reperfusion, blood–brain barrier

## Abstract

Endothelial permeability is a major complication that must be addressed during stroke treatment. Study of the mechanisms underlying blood–brain barrier (BBB) disruption and management of the hypoxic stress-induced permeability of the endothelium following reperfusion are both urgently needed for stroke management. Lysophosphatidic acid (LPA), a bioactive lipid essential for basic cellular functions, causes unfavorable outcomes during stroke progression. LPA-producing enzyme autotaxin (ATX) is regulated in ischemic stroke. We used an electrical cell-substrate impedance sensor (ECIS) to measure endothelial permeability. Mitochondrial bioenergetics were obtained using a Seahorse analyzer. AR-2 probe fluorescence assay was used to measure ATX activity. LPA increased endothelial permeability and reduced junctional protein expression in mouse brain microvascular endothelial cells (MBMEC). LPA receptor inhibitors Ki16425 and AM095 attenuated the LPA-induced changes in the endothelial permeability and junctional proteins. LPA significantly diminished mitochondrial function in MBMEC. ATX was upregulated (*p* < 0.05) in brain microvascular endothelial cells under hypoxic reperfusion. ATX activity and permeability were attenuated with the use of an ATX inhibitor in a mouse stroke model. The upregulation of ATX with hypoxic reperfusion leads to LPA production in brain endothelial cells favoring permeability. Inhibition of the ATX–LPA–LPAR axis could be therapeutically targeted in stroke to achieve better outcomes.

## 1. Introduction

The blood–brain barrier (BBB), a physiological barrier in the microvasculature between the blood vessels and the brain tissue, is essential for maintaining the function of the central nervous system [1]. The BBB keeps neurotoxic plasma-derived components, cells, and pathogens away from the brain [2]. Endothelial cells and their physiological properties aid in limiting vessel permeability and sustaining the BBB [3]. Many cerebral diseases, such as multiple sclerosis, epilepsy, and stroke, share BBB dysfunction as a central element of their pathology [3]. Recently, BBB repair has been proposed as a major therapeutic approach to limit the progression of the pathophysiology of stroke [4,5,6,7]. The mechanisms underlying BBB dysregulation during stroke could be targeted as therapeutics when studied effectively and translated from bench to bed [7]. Junctional proteins are one of the major contributors to BBB formation. Membrane proteins such as occludins, claudins, junction adhesion molecules (JAMs) are tight junctional proteins, and cadherins are adherens junctional proteins [8]. The BBB dynamic regulation and paracellular permeability by these junctional proteins are supported by adapter proteins such as zonula occludens (ZO-1, ZO-2, and ZO-3) and catenins (β-catenin, γ-catenin, and p120-catenin). These proteins are dysregulated and disassembled with the pathological progression of stroke [8]. A better understanding of the mechanisms of BBB disruption during ischemic reperfusion could lead to the formulation of effective therapeutics for stroke.

Lysophosphatidic acid (LPA) is a simple phospholipid that induces various basic cellular responses such as proliferation, migration, platelet aggregation, and cytokine and chemokine secretion [9]. As a widely available bioactive lipid, it is involved in the development of pathological conditions that lead to significant extracellular signaling [10]. LPA causes cellular effects through its six known G-protein coupled receptors (LPAR1–LPAR6) [11]. LPAR1/LPAR2/LPAR3 are endothelial differentiating gene (Edg) family receptors, while LPAR4/LPAR5/LPAR6 are non-Edg or P2Y purine receptors (P2YR). Various intracellular signaling mediators are activated by these receptors, with some receptors having overlapped signaling [10]. It has already been documented that lysophosphatidic acid (LPA) and its receptors are involved in permeability regulation in various tissues such as intestinal epithelial cells [12], corneal epithelial cells [13], lung microvascular endothelial cells [14], and brain microvessel endothelial cells [15]. In many models of brain pathologies, such as intracerebral hemorrhage, cerebral ischemia, and edema, LPA levels have been shown to be elevated, leading to disruption of the BBB [10,16].

LPA is extensively produced in the body by a glycoprotein enzyme called autotaxin (ATX) [17,18]. ATX is a secretory protein that is essential in early development for the formation of the nervous system [19] and blood vessels [17], but it is dispensable in adult life [18]. ATX has been observed to be involved in various pathophysiologies such as pulmonary fibrosis [20], cancer [21], rheumatoid arthritis [22], and liver fibrosis [23]. Earlier evidence revealed ATX to be a strong therapeutic target due to its pathological functions [24]. Nevertheless, ATX and its effect on permeability, specifically during endothelial ischemic-reperfusion, has not been studied enough. Oxidative stress brought about during ischemia-reperfusion causes neuroinflammation with an initial pathological impact on cerebral microvasculature after stroke [25]. This study focuses on the effect of the ATX–LPA–LPAR axis on endothelial permeability following ischemic-reperfusion.

## 2. Results

### 2.1. LPA-Mediated Endothelial Permeability In Vitro

**Figure 1:**
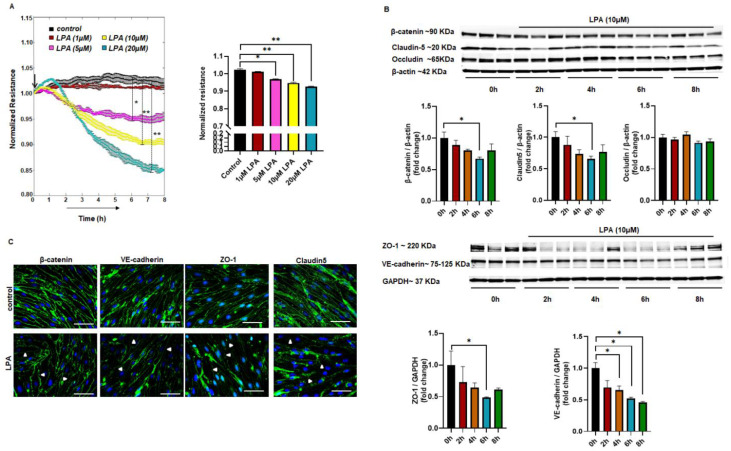
LPA increases permeability and alters junctional proteins in MBMEC. (**A**) Measurement of cell interendothelial permeability using ECIS in MBMEC with control (no LPA) and LPA (1 μM, 5 μM, 10 μM, or 20 μM) treatments. (**B**) Western blotting for protein expression in MBMEC for β-catenin, occludin, claudin-5, ZO-1, and VE-cadherin. (**C**) Immunocytochemistry for ZO-1, β-catenin, claudin-5, and VE-cadherin after 6 h of LPA treatment in MBMEC. White arrow heads show areas of disrupted cell border. Scale bar = 20 μm. All values are mean ± SEM. * *p* < 0.05, ** *p* < 0.01 compared to control using a Mann–Whitney U test.

Mouse brain microvascular endothelial cells (MBMEC) were treated with different doses of LPA (1 µM, 5 µM, 10 µM, or 20 µM). MBMEC monolayers were treated with LPA after 24 h of serum starvation, and the effect was observed for up to 8 h post-LPA treatment. The resistance was measured and analyzed in real-time using ECIS software. As shown by the decreased resistance, LPA caused increased permeability in the MBMEC monolayer. We observed increased permeability in MBMEC monolayers, starting with 5 µM LPA. There was a concentration- and time-dependent increase in permeability (Figure 1A). A significant rise in paracellular permeability following treatment with 10 and 20 µM LPA showed that LPA signals cell contraction in brain microvascular endothelial cells. In addition, when human brain microvascular endothelial cells (HBMEC) were treated as above with different concentrations of LPA, they showed a similar pattern of concentration- and time-dependent increases in paracellular permeability (Figure S1).

### 2.2. Altered Expression of Junctional Proteins with LPA Treatment

Following our observation of enhanced permeability with LPA, we were interested to see the effect of LPA on junctional proteins. The MBMEC monolayer was exposed to 10 µM LPA after 24 h of serum starvation. The cell lysates collected every 2 h were blotted for different junctional proteins: β-catenin, VE-cadherin, occludin, claudin-5, and zonula occludens- 1 (ZO-1). Normalized to GAPDH, the protein expression was downregulated as the time of LPA exposure increased for all of the junctional proteins except occludin. LPA was found to reduce the protein level expression of β-catenin, claudin-5, and ZO-1 following treatment, with a significant reduction demonstrated at 6 h post-treatment compared to controls (Figure 1B). In addition, VE-cadherin showed a significant time-dependent decrease in protein expression compared to the control (Figure 1B). Immunofluorescence analysis of the junctional proteins (ZO-1, β-catenin, VE-cadherin, and claudin-5) was performed at 6 h of exposure. Consistent with the results from Western blotting, there was a downregulation in ZO-1, β-catenin, VE-cadherin, and claudin-5 in the LPA-exposed group compared to the control (Figure 1C), showing that junctional proteins were altered by LPA exposure in MBMEC.

### 2.3. LPA Increases Endothelial Permeability via the LPAR1–ROCK Pathway

**Figure 2:**
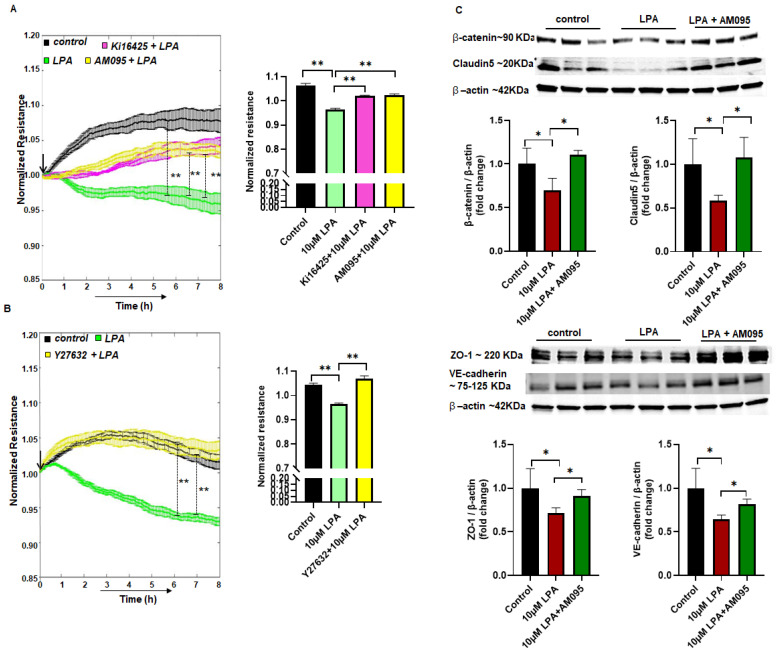
LPA–LPAR1–ROCK causes permeability in MBMEC. (**A**) Measurement of cell interendothelial permeability using ECIS in MBMEC with control (no LPA), LPA (10 μM), Ki16425 (10 μM), or AM095 (10 μM) treatments. (**B**) Measurement of cell interendothelial permeability using ECIS in MBMEC with control (no LPA), LPA (10 μM), or Y27632 (10 μM) treatments. (**C**) Western blotting for protein expression in MBMEC for β-catenin, claudin-5, ZO-1, and VE-cadherin with control (no LPA), LPA (10 μM), or AM095 (10 μM) treatments. All values are mean ± SEM. * *p* < 0.05, ** *p* < 0.01 compared to control using a Mann–Whitney U test.

To identify the downstream signal of LPA pertaining to the permeability, we used the LPAR1/LPAR2/LPAR3 receptor inhibitor Ki16425 and LPAR1 receptor inhibitor AM095. The MBMEC monolayer was pre-treated with 10 µM Ki16425 or 10 µM AM095 1 h before exposure to LPA. Both Ki16425 and AM095 significantly attenuated the paracellular permeability as compared to the effect on permeability caused by LPA (Figure 2A). The Rho pathway is well known for cell contraction in endothelial cells, so we used the ROCK1/ROCK2 inhibitor Y-27632 with LPA treatment for permeability signaling. Y-27632 (10 µM) pre-treatment for 1 h was able to significantly prevent the paracellular permeability effect caused by LPA (Figure 2B), suggesting that LPA increases permeability in MBMEC via the LPAR1–ROCK pathway. With Western blotting, we observed that AM095 also rescued the β-catenin, claudin-5, ZO-1, and VE-cadherin protein degradation compared to the LPA treatment in MBMEC (Figure 2C). This suggests that the LPAR1 receptor inhibitor could be effective against the permeability caused by LPA.

### 2.4. Increased Levels of Superoxide and Reduced Mitochondrial Bioenergetics in LPA-Treated MBMEC

**Figure 3:**
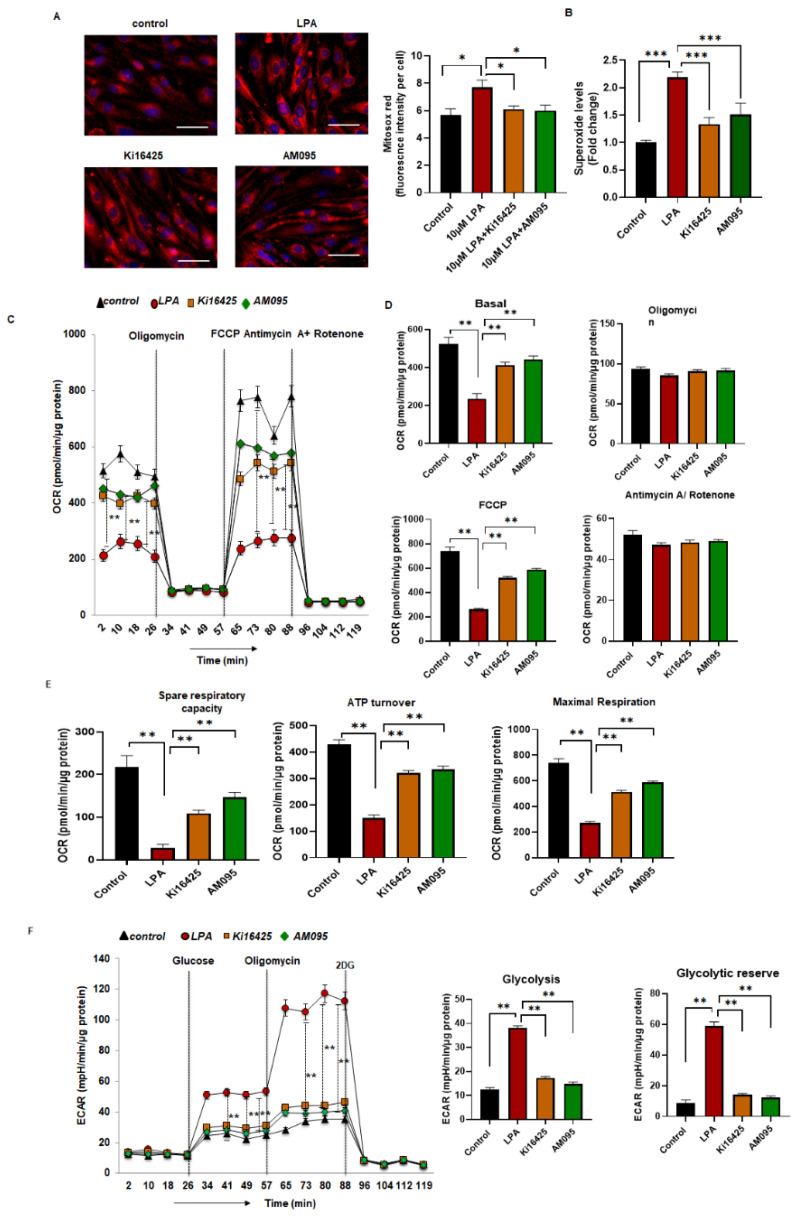
Increased levels of superoxide and reduced mitochondrial bioenergetics in LPA-treated MBMEC. (**A**) Representative fluorescence imaging for Mitosox Red dye in MBMEC with control (no LPA), LPA (10 μM), Ki16425 (10 μM) or AM095 (10 μM) treatment for 4 h quantified in fluorescence intensity measured per cell. Scale bar = 10 μm. (**B**) Superoxide measured using HPLC in MBMEC after staining with a dihydroethidium fluorescence probe for control (no LPA), LPA (10 μM), Ki16425 (10 μM), or AM095 (10 μM) for 4 h. (**C**,**D**), Graph represents OCR (pmol/min/μg protein) measurements in MBMEC treated with control (no LPA), LPA (10 μM), Ki16425 (10 μM) or AM095 (10 μM) at baseline and after sequential addition of oligomycin, FCCP, and antimycin A+rotenone. (**E**) Spare respiratory capacity, ATP turnover, and maximum respiration values analyzed in MBMEC treated with control (no LPA), LPA (10 μM), Ki16425 (10 μM) or AM095 (10 μM) for 4 h. (**F**) ECAR (graph and quantification) measured in MBMEC treated with control (no LPA), LPA (10 μM), Ki16425 (10 μM) or AM095 (10 μM) for 4 h represented by analyzed glycolysis and glycolytic reserve graph. All values are mean ± SEM. * *p* < 0.05, ** *p* < 0.01, *** *p* < 0.001, compared to control using Student’s *t*-test for two groups and ANOVA followed by the Bonferroni post hoc test for more than two groups.

To further elucidate the mechanism underlying the LPA-induced increase in permeability, we measured the effect of LPA on the mitochondria of endothelial cells. Mitosox Red staining and high-performance liquid chromatography (HPLC) were used to detect superoxide levels following LPA exposure. Mitosox Red staining for mitochondrial superoxide showed significantly increased levels of superoxide in MBMEC treated with LPA compared to levels in the control cells. The fluorescence intensity in the groups was quantified per cell (Figure 3A). With Ki16425 and AM095 treatment, the mitochondrial superoxide levels were significantly alleviated. Superoxide was measured in MBMEC using HPLC with dihydroethidium (DHE) dye. Following treatment with LPA, there was a 2-fold increase in the levels of superoxide at 4 h of LPA exposure compared to control (Figure 3B). The use of the inhibitors Ki16425 or AM095 for a 1 h pre-treatment significantly reduced the superoxide levels compared to LPA treatment (Figure 3B).

Next, mitochondrial bioenergetics were measured using a Seahorse analyzer. MBMEC were treated with control, LPA, and LPA with Ki16425 or AM095 prior to oxygen consumption rate (OCR) measurement. LPA decreased OCR in MBMEC, whereas Ki16425 and AM095 were able to significantly rescue the basal OCR, spare respiratory capacity, ATP turnover, and maximal respiration (Figure 3C–E) compared with those in LPA-treated MBMEC. During an injury, cells shift their energy production to glycolysis; the extracellular acidification rate (ECAR) measurement represents glycolysis taking place in cells. In MBMEC treated with LPA, there was a significant increase in glycolysis and glycolytic reserve, whereas pre-treatment with Ki16425 and AM095 abolished this effect (Figure 3F). The significant decrease in glycolysis and glycolytic reserve caused by Ki16425 and AM095 treatment suggests that the cells were more efficient with mitochondrial energy production than the cells that received LPA treatment only. These results suggest that LPA affects MBMEC mitochondrial status by compromising its function.

### 2.5. Elevated Expression of ATX with Oxygen Glucose Deprivation–Reoxygenation (OGDR)

**Figure 4:**
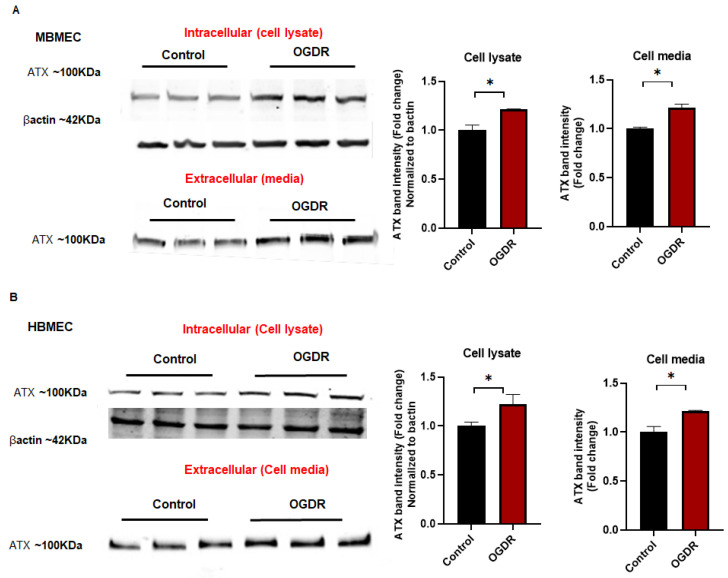
Elevated ATX with OGDR in brain endothelial cells. (**A**) Western blotting for protein expression in MBMEC for ATX with control and ODGR (intracellular and extracellular). (**B**) Western blotting for protein expression in HBMEC for ATX with control and ODGR (intracellular and extracellular). All values are mean ± SEM. * *p* < 0.05, compared to control using a Mann–Whitney U test.

ATX expression was measured in MBMEC with OGDR. The protein expression of ATX was measured with Western blotting. With OGDR, ATX was significantly elevated in MBMEC (Figure 4A) and HBMEC (Figure 4B). In addition, we measured intracellular ATX in cell lysate, and extracellular ATX in cell-conditioned media. These observations demonstrated that ATX is upregulated in brain vascular endothelial cells, during ischemic-reperfusion leading to increased LPA production.

### 2.6. ATX Inhibitor Reduces Permeability in a Mouse Stroke Model

**Figure 5:**
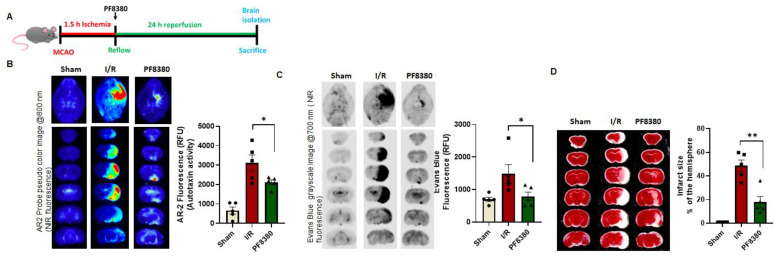
ATX inhibitor reduces permeability and infarct volume in ischemic stroke. (**A**) MCAO was performed in mice followed by 1.5 h ischemia and 24 h reperfusion. PF8380 was administered right after reflow. (**B**) Enzymatic activity for ATX measured with AR-2 fluorescence and quantified as relative fluorescence units (RFU) in sham, I/R, and PF8380 mouse brains. (**C**) Evans Blue fluorescence measured and quantified as relative fluorescence units (RFU) in sham, I/R, and PF8380 mouse brains. (**D**) TTC staining of mouse brain slices and respective infarct volume (% of the hemisphere) measured in sham, I/R, and PF8380 mouse brain slices. All values are mean ± SEM (n = 5). * *p* < 0.05, ** *p* < 0.01, compared to I/R using a Mann–Whitney U test.

Following the observation that ATX expression increased in brain endothelial cells, the ATX inhibitor was used in mice post- ischemia reperfusion (I/R). Post-treatment using an inhibitor is more clinically relevant, so PF8380 was injected during the reflow (Figure 5A). The ATX activity in the brains of mice treated with PF8380 was significantly alleviated compared to mice who underwent I/R only (Figure 5B). Furthermore, permeability measured using Evans Blue in mouse brains showed that post-treatment with PF8380 considerably reduced the permeability in the brains of these mice compared to those in the I/R only group (Figure 5C).

Infarct size was also measured in the mouse stroke model using an ATX inhibitor and it was observed that infarct size was reduced following PF8380 treatment (Figure 5D) compared to results in the mice in the I/R only group. These findings advance the concept that ATX is dysregulated in the stroke model with increased expression in endothelial cells, ultimately increasing the vascular permeability.

## 3. Discussion

Limiting BBB disruption is a significant challenge in controlling pathological progression in stroke [26]. Microvascular endothelial cells are the primary cells responsible for regulation of blood–brain transport and maintaining the barrier between the brain and the blood [2]. Disruption of this dynamic process increases pathology following ischemic stroke. Currently, most studies have focused on evaluating BBB injury and progression mechanisms, as neither is entirely understood [27]. BBB protection could be a prolific therapeutic target upon robust investigation [28]. Earlier findings suggest that LPA regulates permeability in various organs, such as the lungs [29], intestine [12], and brain [15] via modulation of its receptors. We have previously shown that LPA is upregulated with cerebral ischemia-reperfusion [16], which led us to anticipate permeability due to LPA in the brain. So, having investigated LPA upregulation in stroke [16,30], we were interested in exploring the effect of LPA on the BBB and endothelial junctional proteins using MBMEC as an in vitro model. Using different concentrations of LPA treatment in MBMEC, we observed that permeability was increased in a dose- and time-dependent manner. When treated with LPA, HBMEC also showed a similar pattern (Figure S1). Our findings are in accord with those of Sarkar et al. [31] and On et al. [15], which also showed that LPA could acutely increase the permeability in the cerebrovasculature. The increase in permeability could be due to the internalization or degradation of junctional proteins [32]. Using Western blot analysis, we demonstrated that LPA treatment at 6 h significantly reduced β-catenin, claudin-5, and ZO-1 expression levels in MBMEC. The levels of VE-cadherin were observed to have decreased even at an earlier time point. This suggests that these junctional proteins were degraded upon exposure to LPA. Immunocytochemistry analysis of β-catenin, claudin-5, ZO-1, and VE-cadherin in LPA-treated MBMEC also revealed a disrupted border between endothelial cells. It has been documented that claudin-5 is targeted for degradation by matrix metalloproteinase after an ischemic insult, and the loss of claudin-5 is responsible for the disruption of the BBB in ischemic stroke [33,34]. Earlier findings suggest that LPA enhances matrix metalloproteinase expression in mRNA, protein levels, and also the enzymatic activity of endothelial cells [35,36]. We did not observe a statistically significant decrease in occludin with LPA exposure, suggesting that it may be internalized rather than degraded. Wang et al. also detected an internalization phenomenon for occludin, which contributed to the disruption of the epithelial barrier in CaCo-2 cells [37]. A well-known feature of junctional proteins is they are degraded and regulated by endocytosis [32] aided by Rho/ROCK-mediated actin contraction [38]; it is possible that LPA receptors also mediate the downregulation of junctional proteins. LPAR1 affects cell–cell interaction and adhesion [39,40], so we pre-treated MBMEC with Ki16425 or AM095 before exposure to LPA. MBMEC exposed to LPA showed decreased permeability following Ki16425 or AM095 pre-treatment. The expression of junctional proteins (β-catenin, claudin-5, ZO-1 and VE-cadherin) was also rescued by AM095 pre-treatment. As LPAR1 acts through the Rho–ROCK pathway [40] with the use of Y27632, permeability in MBMEC was significantly rescued. The ROCK pathway is involved in expressing proteolytic enzymes, matrix metalloproteinase, and urokinase-type plasminogen activator (uPA), leading to LPA-induced BBB disruption [36]. These observations suggest that LPA increases permeability through the LPA–LPAR1–ROCK pathway in MBMEC. Our findings are in accord with those of Amerongen et al. [41], which demonstrated LPA-induced cytoskeletal reorganization through the ROCK pathway.

In addition to causing disruption of the BBB in brain endothelial cells, LPA was also observed to compromise mitochondrial function and redox balance. Mitosox Red fluorescence was elevated in MBMEC following LPA treatment, as were the levels of superoxide. LPA administration has been shown to generate ROS through NADPH oxidase 4 [42], and ROS is a second messenger for LPA-mediated signaling. Pre-treatment with Ki16425 or AM095 reduced the levels of superoxide, demonstrating that LPAR1 inhibitors can reduce the oxidative stress caused by LPA in endothelial cells. Previously, Klomsiri et al. [43] and Chabowski et al. [44] also demonstrated oxidative stress induction by LPA via increasing H_2_O_2_ through LPAR1. Chabowski et al. [44] showed that through LPAR1, LPA induced mitochondrial ROS (H_2_O_2_-mitochondria-derived hydrogen peroxide) in adipose arterioles. Like Chabowski et al., we also observed no expression of LPAR3 (Figure S2) in our MBMEC, signifying that LPAR1 plays a major role in the vasculature by relaying the signal by which LPA induces ROS. Oxidative damage elicits a mitochondrial stress response in the endothelial cells triggering mitochondrial impairment. The mitochondrial bioenergetics were diminished following LPA exposure in MBMEC. LPA lowered basal OCR and decreased spare respiratory capacity, ATP turnover, and maximal respiration in these cells. When treated with Ki16425 and AM095, the mitochondrial bioenergetic profile was significantly revived. The glycolysis and glycolytic reserve measured with ECAR was increased following treatment with LPA, but Ki16425 and AM095 treatment halted the trend towards an increase in glycolysis. These findings suggest that inhibition of LPA or its receptors limits the mitochondrial damage caused by LPA. Previously, it has been shown that mice with a heterozygous ATX deficiency (ATX+/−) were associated with improved mitochondrial energy metabolism when exposed to a high-fat, high-sucrose diet [45]. Recently, we showed that cardiomyocyte cells exposed to LPA demonstrated decreased mitochondrial oxidative phosphorylation [46].

The regulation of ATX during hypoxia has not been explicitly studied in endothelial cells. Paracrine/autocrine signaling of ATX is a well-known mechanism [47,48]. In our study, for the first time, we show that increased ATX expression with OGDR in brain endothelial cells could play a vital role in increasing permeability in its vicinity by elevating localized LPA production. The hypoxic microenvironment could play a favorable role in inducing ATX expression, and ultimately, LPA formation affecting the BBB. Earlier, a study by Farquhar et al. [49] showed that ATX expression was increased with hypoxia in Huh-7 cells (epithelial-like, tumorigenic cells) through hypoxia-inducible factors (HIFs). The signaling mechanism for the ATX increase in brain endothelial cells could also be mediated by HIFs, as HIFs increase during hypoxia and regulate various downstream signaling molecules [50,51]. A further detailed investigation will be required to identify the mechanistic regulation of ATX during hypoxic reperfusion. As ATX was elevated in the endothelial cells, an ATX inhibitor was used post-surgery in a mouse ischemic stroke model, making it more clinically translational. This showed that treatment with the ATX inhibitor eased pathological events after stroke, including alleviation of the vascular permeability and reduced infarct size in mouse brains. Both the events were directly correlated with the decrease in ATX activity achieved with the use of an inhibitor, suggesting that ATX plays a negative role by elevating BBB disruption through LPA production in the stroke-induced brain microenvironment. We have also shown previously [16] that ATX activity is increased during I/R and that pre-treatment (before surgery) with an ATX inhibitor reduces the stroke progression by decreasing LPA. It has previously been observed that stroke increases LPA concentration in mice [16,30]. Our observations strongly support the idea that the ATX–LPA axis plays a major role in the vascular permeability brought about by perfusion following stroke. Endothelial-specific ATX could play a significant role in ATX concentration in the infarct region, as suggested by the in vitro data in this study. This is also supported by our earlier work [16], where LPA was found to be elevated in the vasculature of mouse brains following I/R, suggesting negative effects caused by the endothelial ATX–LPA axis in ischemic-reperfusion.

## 4. Methods

### 4.1. Animal Models of Ischemic Stroke

All the procedures and protocols for animal studies were approved by the Institutional Animal Care and Use Committee (IACUC) at LSU Health Sciences Center–Shreveport. The protocols were developed in accordance with the NIH guide for Care and Use of Laboratory Animals. Three-month-old male C57BL/6J mice were used for ischemia-reperfusion (I/R) surgery or sham surgery. Transient middle cerebral artery occlusion was performed in mice with 1.5 h of ischemia followed by 24 h of reperfusion. In mice, the right common and external carotid arteries were exposed and ligated. The right middle cerebral artery was occluded with silicone rubber-coated monofilament MCAO suture (Doccol Corporation; Sharon, MA, USA) to induce ischemia; sutures were later withdrawn to induce reperfusion. The monofilament was advanced from the external carotid artery into the internal carotid artery and then to the point where the middle cerebral artery (MCA) branches off from the internal artery. Isoflurane (induction at 3% and maintenance at 1.5%) mixed with 30% O_2_/70% N_2_ gas administered via facemask was used to anesthetize mice. A temperature-controlled heating pad (Harvard Apparatus, Germany) was used to maintain the body temperature of the mice at 37 °C throughout the surgery. A laser-Doppler flow probe (PF 5010 LDPM Unit; PERIMED, Sweden) was used to observe the regional blood flow through the MCA in the mice. The sham group had surgery but not occlusion of the MCA. Carprofen (2 mg/kg) was administered subcutaneously post-surgery. Mice were observed for 1 h post-surgery and kept on a heating pad to prevent adverse effects. Soft food and clean bedding were in place for the mice. Twenty-four hours following reperfusion, mice were sacrificed using absolute isoflurane exposure and perfused with saline before isolating the brains for experiments. For the measurement and quantification of infarct size in mouse brains, brain slices of 1.5 mm were prepared and stained with triphenyl tetrazolium chloride (TTC) for 30 min. ImageJ software was used to quantify the infarct area.

### 4.2. Cell Culture

Brain microvascular endothelial cells represent acceptable in vitro models to pursue signaling studies in BBB. So, mouse brain microvascular endothelial cells (MBMEC) (C57-6023, Cell Biologics) and human brain microvascular endothelial cells (HBMEC) (HBEC-5i, ATCC) were used in our study to evaluate the effect of LPA. Cells were propagated in a growth medium containing essential and non-essential amino acids, vitamins, organic and inorganic compounds, hormones, growth factors, and trace minerals, and supplemented with endothelial cell growth supplement, antibiotics, and fetal bovine serum. All cells were maintained at 37 °C in a humidified atmosphere of 5% CO_2_.

### 4.3. mRNA Expression of LPA Receptors

Total RNA was extracted from MBMEC using the Purelink RNA mini kit (Invitrogen, Waltham, MA, USA) following the manufacturer’s instructions. cDNA was prepared with Multiscribe reverse transcriptase enzyme (4368814 High-Capacity cDNA Archive Kit; Applied Biosystems, Foster City, CA, USA), and mRNA expression was measured with RT-PCR reaction using TaqMan gene expression assays and TaqMan Universal PCR Master Mix (4444556 Applied Biosystems, Foster City, CA, USA) on a Viaa7 Real-Time PCR System (Applied Biosystems, Foster City, CA, USA). Receptor expression analysis was done using the ∆ct method normalized to glyceraldehyde-3-phosphate dehydrogenase (GAPDH). Primers used were Mm01346925_m1 (LPAR1), Mm00469562_m1 (LPAR2), Mm00469694_m1 (LPAR3), Mm02620784_s1 (LPAR4), Mm02621109_s1 (LPAR5), Mm00613058_s1 (LPAR6), and Mm99999915_g1 (GAPDH) from Applied Biosystems.

### 4.4. Superoxide Measurement

A high-performance liquid chromatography (HPLC) system coupled with UV-vis and fluorescence detectors was used to measure superoxide in brain tissue and MBMEC. Medium containing 10 µM DHE was added to the cells for superoxide measurement in cells, followed by incubation for 30 min. The medium was removed, and the cells were scraped into 1 mL ice-cold DPBS. The cell lysate was transferred to a 1.5 mL tube and centrifuged at 1000× *g*, 4 °C for 5 min. The excess DPBS was aspirated, and following the addition of 200 μL 0.1% Triton X-100 in DPBS, the cells were lysed by sonication followed by centrifugation at 10,000× *g*, 4 °C for 5 min. The lysate supernatant was used for protein estimation; 100 μL was added to a tube containing 100 μL MeOH/HClO_4_ solution. Protein was precipitated by incubating the tube on ice for 2 h. The supernatant was obtained by centrifugation of the tubes at 12,000× *g*, 4 °C for 10 min. An aliquot of 100 μL supernatant was added to 100 μL 1 M phosphate buffer (pH 2.6). Excess buffer was removed following centrifugation at 12,000× *g*, 4 °C for 5 min. The supernatant was transferred to a new tube for HPLC superoxide measurement. Inhibitors (Ki16425 (10 μM) and AM095 (10 μM)) were added 1 h prior to LPA treatment.

### 4.5. Mitochondrial Bioenergetics

As the state of the mitochondria reflects the physiological state of the cells, mitochondrial bioenergetics were measured in MBMEC. The OCR (oxygen consumption rate) and ECAR (extracellular acidification rate) were analyzed using a Seahorse extracellular flux analyzer (XF-24, Seahorse Biosciences, Chicopee, MA, USA). For cell mitochondrial bioenergetics analysis, MBMEC were grown to confluence in a Seahorse plate and treated with control (no LPA), LPA, or LPA with inhibitors. The real-time bioenergetic activity of the cells or mitochondria and the effects of treatments were observed as free protons, and the concentration of oxygen was measured using the XF-24. OCR and ECAR values were quantified in pmol/min/μg, and mpH/min/μg, respectively, with normalization, carried out with respect to the total protein content. The initial basal value of OCR is interrupted by the addition of oligomycin (Complex V inhibitor), giving values for ATP-linked OCR. FCCP (an uncoupler) and rotenone + antimycin (Complex I and Complex III inhibitor) addition determines the maximal OCR capacity and spare OCR capacity, respectively. For ECAR experiments, a glucose-free medium was used. Following the sequential addition of glucose (25 mM), oligomycin (1 μg/mL), and deoxyglucose (25 mM), we measured the rate of glycolysis and glycolytic reserve in MBMEC. Inhibitors (Ki16425 (10 μM) and AM095 (10 μM)) were added 1 h prior to LPA treatment.

### 4.6. Transendothelial Electrical Resistance (TEER)

TEER was determined using an electrical cell-substrate impedance sensor (ECIS) ΖΘ System (Applied Biophysics, Troy, NY, USA). MBMEC were seeded at 10^5^ cells per well into gelatin-coated chambers of ECIS arrays and allowed to attach and form a confluent monolayer. Basal endothelial media with 0.1% delipidated serum with control (no LPA), LPA, or LPA with inhibitors were used for the treatments. A 1 μA AC signal at 4 kHz was applied. Total impedance was reported by monitoring the voltage across the electrodes and its phase relative to the applied current. The cell-covered electrode unit was treated as an RC circuit, from which impedance data were later converted into monolayer resistance representing barrier function. Normalized resistance data obtained from the ECIS software were plotted for graphs.

### 4.7. Western Blot Analysis

MBMEC were treated with control, LPA, or LPA with inhibitors and washed with PBS following treatments. The cell lysate was prepared in ice-cold RIPA lysis buffer (Pierce Ripa buffer, ThermoFisher, Waltham, MA, USA). The cell lysate was centrifuged at 12,000× *g*, 4 °C for 15 min to obtain the supernatant used for protein estimation. SDS-PAGE was performed with 40 µg of total protein per well. After SDS-PAGE, the proteins were transferred to a PVDF membrane and visualized using an LI-COR Odyssey (LI-COR Biosciences, Lincoln, NE, USA). Inhibitors were added 1 h prior to LPA treatment. For blotting protein from the media, cell-conditioned media was collected and concentrated using protein concentrator (Amicon UFC903024, UFC905024, Sigma, St. Louis, MA, USA). Then, SDS PAGE was performed and the protein was transferred to a PVDF membrane. For quantification, the media protein was normalized to total protein from ponceau staining. The primary antibodies incubated overnight were rabbit anti-ZO-1 (61-7300, ThermoFisher, Waltham, MA, USA), mouse anti-claudin-5 (35-2500, Invitrogen, Waltham, MA, USA), rabbit anti-VE-cadherin (ab205336, Abcam, Cambridge, MA, USA), rabbit anti-β-catenin (ab32572, Abcam) rabbit anti occludin (ab216327, Abcam) and mouse ATX (ab77104, Abcam). Secondary antibodies, goat anti-mouse 800 and goat anti-rabbit 700 (Licor Biosciences), were used for 2 h at room temperature. The membranes were imaged using Licor Odyssey infrared imager and quantified using the Licor software.

### 4.8. Immunofluorescence Staining of Junctional Proteins

For immunofluorescence staining, MBMEC were cultured on chambered slides. After incubating with or without LPA (10 μM) media, the cells were fixed with 4% paraformaldehyde and permeabilized using 0.2% Triton X-100. The cells were blocked with 10% goat serum for 1 h, then incubated overnight at 4 °C with mouse anti-ZO-1 (ThermoFisher), mouse anti-claudin-5 (Invitrogen), rabbit anti-VE-cadherin (Abcam), and rabbit anti-β-catenin (Abcam) as primary antibodies. The cells were incubated with the following secondary antibodies: AlexaFluor 488 goat anti-mouse (ThermoFisher), AlexaFluor 488 goat anti-rabbit (ThermoFisher), or AlexaFluor 594 goat anti-rabbit (ThermoFisher) for 1 h at room temperature. The cells were mounted with a vecta shield mounting medium with DAPI (H-1500, Vector Laboratories, Burlingame, CA, USA) and visualized using a fluorescence microscope with a mounted Nikon camera.

### 4.9. Mitosox Red Mitochondrial Superoxide Staining

MBMEC were cultured on chambered slides (154526, ThermoFisher). The cells were treated with control or 10 µM LPA for 4 h. For the last 10 min, 5 µM Mitosox Red (M36008, ThermoFisher) was added to the medium. Cells were immediately fixed with 4% paraformaldehyde and mounted with a Vecta shield mounting medium with DAPI. The slides were then visualized using a fluorescence microscope with a mounted Nikon camera.

### 4.10. OGDR (Oxygen Glucose Deprivation–Reoxygenation)

OGD or hypoxia was induced by replacing the initial culture medium with no-glucose endothelial medium (GPF1168, Cell Biologics). MBMEC/HBMEC were incubated in an air-tight chamber gassed with pure N_2_ at 37 °C for 4 h. Following exposure to hypoxia, the medium was then replaced with Opti-MEM culture medium, and the cells were reoxygenated for 24 h in a humidified tissue culture incubator with 5% CO_2_-95% air.

### 4.11. ATX Activity Assay and Evans Blue Permeability Assay

The ATX activity was measured in brain and brain slices using the AR-2 probe, a fluorogenic analog of LPC [52]. In vivo measurement and quantification of ATX activity was performed as in Bhattarai et al. [16] with modifications. Briefly, AR2 (0.5 mg/kg) was given retro-orbitally 3 h before the mice were sacrificed. The mice were perfused transcardially, and the brains were removed for imaging. The brain slices (1.5 mm) are imaged and quantified using a near-infrared imager (NIR) LI-COR Odyssey (LI-COR Biosciences) at 800 nm. For the PF8380 group, the inhibitor was injected (30 mg/kg body weight) [53] intraperitoneally at the time of reperfusion. Brain vascular permeability was acquired by measuring the fluorescence intensity of Evans Blue, as it is a fluorophore [54]. Mice were injected intraperitoneally with 1.2 mL/kg (0.1 mL of a 1% solution in PBS) Evans Blue 3 h before isolating the brain for imaging. The fluorescence intensity was measured and quantified at 700 nm in the NIR imager.

### 4.12. Statistical Analysis

Data analysis was conducted using Graphpad Prism 8. Data are reported as means ± standard error of mean (SEM), with all experiments carried out with a minimum of three replicates. One-way analysis of variance (ANOVA) followed by the Bonferroni post hoc test or the unpaired Student’s *t*-test (Mann–Whitney U test: non-parametric *t*-test to compare whether there is a difference in the dependent variable for two independent groups) was used to identify significant differences between groups. A *p*-value of less than 0.05 was considered significant.

## 5. Conclusions

Briefly, the following observations were made. First, LPA negatively regulates endothelial permeability by decreasing the expression of the junctional proteins in brain endothelial cells. In addition, the LPA–LPAR1–ROCK pathway is involved in the increased permeability in brain endothelial cells, mitochondrial function is also adversely affected by LPA treatment in brain endothelial cells, and hypoxic reperfusion leads to an elevation in ATX expression in these cells. Finally, ATX activity directly correlates to increased permeability and infarct size in the mouse stroke model. In conclusion, this study suggests that the endothelial ATX–LPA axis plays a crucial role in ischemic reperfusion. Furthermore, since it has been established that ATX is essential [55] during the early developmental stages but not in adulthood [18], the ATX–LPA axis is a safe choice for a therapeutic target to manage stroke outcomes.

## Data Availability

Not applicable.

## Supplementary Materials

The following supporting information can be downloaded at: https://www.mdpi.com/article/10.3390/ijms23084138/s1.
