# Supplementary material for: The ATX–LPA Axis Regulates Vascular Permeability during Cerebral Ischemic-Reperfusion
Source: Int J Mol Sci. 2022 Apr 8;23(8):4138. doi: 10.3390/ijms23084138 (PMC9024554; doi:10.3390/ijms23084138)
Supplement: Supplementary file 1 [file ijms-23-04138-s001.zip › ijms-1654251-supplementary.pdf]

Fig S1

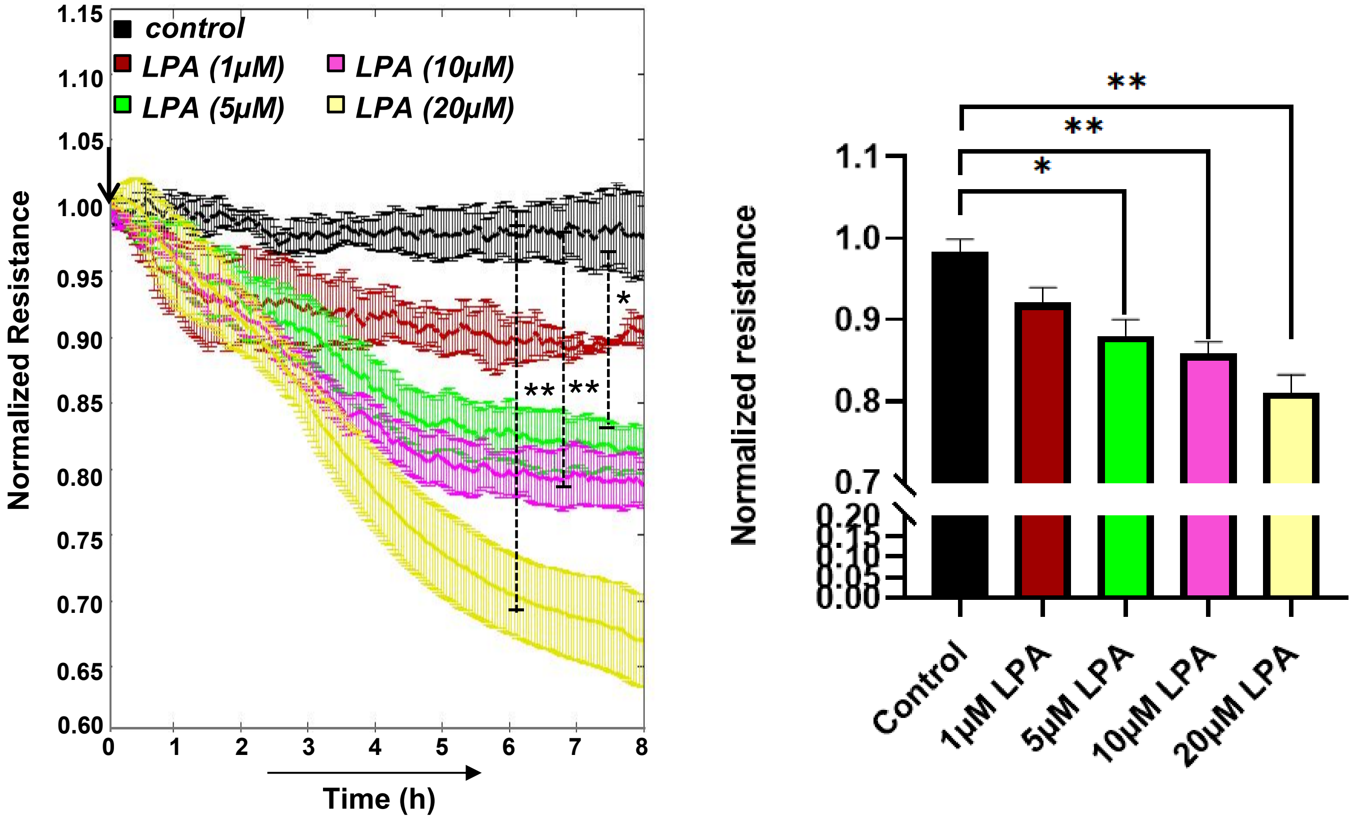

Supplementary Figure S1. Measurement of cell inter-endothelial permeability using ECIS in HBMEC with control (no LPA) and LPA (1 µM, 5 µM, 10 µM, or 20 µM) treatments. All values are mean ± SEM (n=4). *P<0.05, **P<0.01, one-way analysis of variance (ANOVA) followed by the Bonferroni post hoc test.

Fig S2

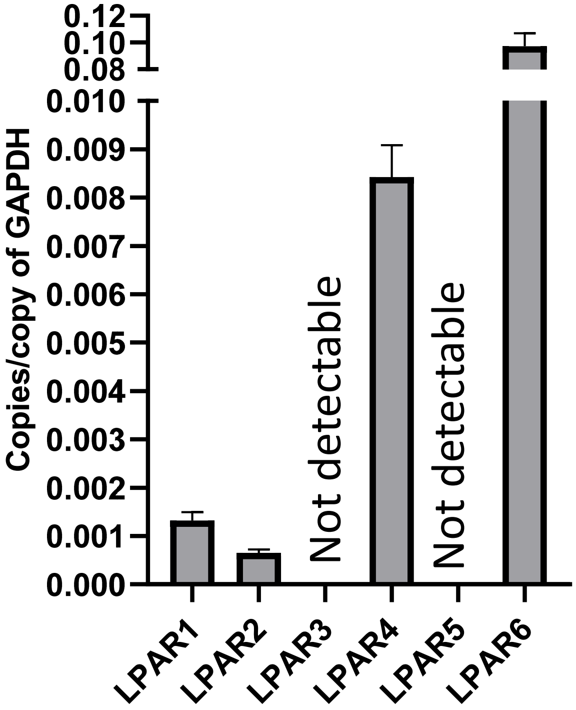

Supplementary Figure S2. LPA receptor mRNA expression was measured in MBMEC. The copies of the receptor's expression were normalized to a copy of GAPDH to plot the graph. All values are mean ± SEM (n=5).
